# Exploring Comfort and Entertainment: The Impact of Campus Design on Student Social Interaction — Insights from Bahrain

**DOI:** 10.12688/f1000research.167517.1

**Published:** 2025-09-04

**Authors:** May Al Saffar

**Affiliations:** 1 Architecture and Interior Design, Ahlia University, Manama, Capital Governorate, Bahrain

**Keywords:** Spatial Design, Social Interaction, Student Engagement, Learning environments, Ahlia University, Bahrain

## Abstract

**Background:**

This study investigates the influence of spatial design elements on social interactions among students at Ahlia University in Bahrain. It emphasizes that well-designed environments can enhance social bonds by exploring how natural and artificial physical features support relationships on campus.

**Methods:**

Utilizing a mixed-methods approach which encompasses on-site observations, student surveys, and space syntax analysis—the study systematically identifies key factors influencing interpersonal attraction and interaction types, including similarity, familiarity, and physical proximity.

**Results:**

The findings demonstrate that students primarily congregate in the cafeteria and communal areas; however, perceptions of comfort and entertainment are significantly diminished owing to insufficient seating and limited recreational facilities. This underscores the considerable influence of the physical environment on influencing student experiences and engagement.

**Conclusion:**

This research demonstrates how considerate spatial planning can foster community development and enhance social cohesion. To elevate campus life, it is advisable to incorporate user feedback into the design process, apply space syntax principles to optimize layout efficacy, and enhance safety and comfort measures. Addressing these aspects can enable universities to create more inviting and functional environments that encourage meaningful interactions and cultivate a robust sense of community among students.

## Introduction

Time has shown many examples of well-designed spaces that have earned the distinction of being “places”. One of the essential goals for the most well-designed spaces is to create opportunities for social interaction in various forms. The greater the chance for social interaction, the more diverse relationships are likely to form ( Sime, 1986). This social connectedness is then fostered, leading to a stronger sense of community and belonging. Belonging to a community is a powerful motivator in life and a catalyst for happiness and overall well-being. Consequently, this sense of belonging is transferred from the community to the place ( Michalski et al., 2020; Zahnow, 2024).

Every built environment has its own identity and characteristics. However, social interaction is vital in all settings, including educational environments. Expanding the network of relationships and making more connections with others is one of the main goals for most university students. University campuses offer three important catalysts for interaction: similarity, familiarity, and physical proximity ( Hurst et al., 2013; Jansz et al., 2021).

The study aims to uncover the impact of spatial design elements on university campuses and their effect on student social interaction. It employs on-site observation, student questionnaires, and space syntax analysis to achieve its objectives. The research was conducted at Ahlia University, Bahrain.

## Background: Physical and Social experience

### Social interaction

Interpersonal Attraction, or liking, is a natural process in which people approach and interact with each other after forming a positive attitude toward one another ( Clark & Mills, 1979). Research reveals that personal characteristics aren’t the only factors that influence interpersonal attraction ( Hurst et al., 2013). Other key factors, such as similarity, familiarity, and physical proximity, also play a significant role. These factors are often interconnected and can be influenced by the physical environment, as noted by Sears et al. (1991) and Jansz et al. (2020) Similarity occurs when people share common interests, backgrounds, goals, or personalities. Familiarity, by contrast, develops through repeated exposure to someone, which increases our preference for and liking of that person ( Kensington-Miller, 2018). People who live, study, or work together are often exposed to the same individuals due to their shared physical proximity. Additionally, interpersonal attraction can be measured consistently across three key dimensions: social (liking someone as a companion), physical (their appearance), and task (their ability to work together or competence) ( McCroskey & McCain, 1974).

Interactions between strangers can be divided into two types: passive and active. Passive interactions are unplanned and unintentional encounters between people ( Waggoner et al., 2009). Active interactions, on the other hand, involve a conversation between two or more people and occur on multiple levels, evolving and changing over time.

**Figure 1.**
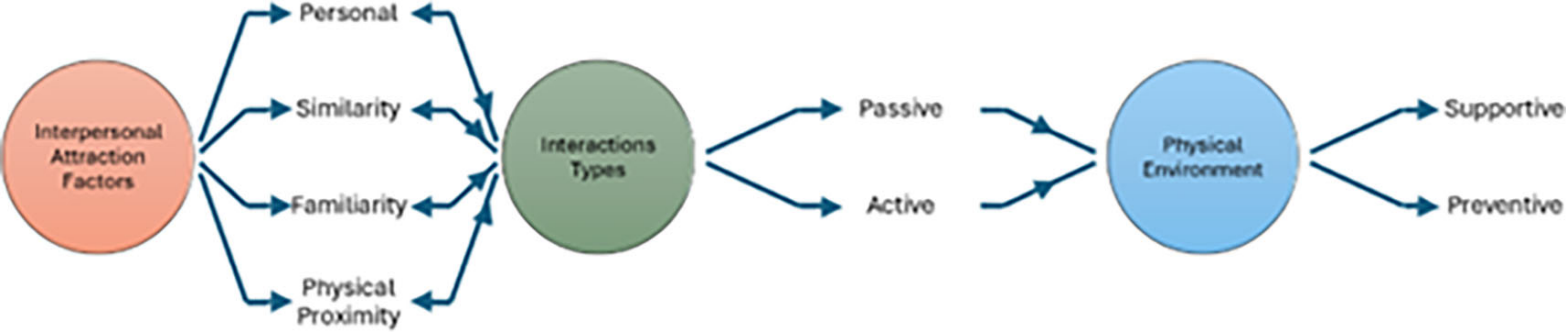
The connection between factors of interpersonal attraction, types of interaction, and the physical environment. Source: Author.

The repeated passive interactions can be transformed into active ones, based on the three previously explained concepts of similarity, familiarity, and physical proximity, as stated by Iachini et al. (2016), and Veranic et al. (2025) Who suggests that opportunities for contact, proximity to others, and the appropriate space to interact enhance the possibility for social interaction, as shown in Figure 1.

At colleges where students concentrate on one subject or field, they often have related areas of study, which fosters a sense of community. Since students interact daily, they get to know each other well, and being on the same campus means they’re nearby, making it easy for them to form connections.

By offering chances to interact and creating a physical environment that supports engagement, students will be encouraged to connect frequently and regularly. More opportunities for social interaction led to the formation of more diverse relationships, which in turn strengthen social connections and foster a greater sense of community and belonging. This is believed to be a powerful motivator for life, happiness, and overall well-being ( Waggoner et al., 2009).

### The relationship between humans and space

Environmental designers assert that architecture can facilitate social interaction. When considering architecture with this objective in mind, it is necessary to evaluate spatial configuration. The spatial configuration is assessed based on the physical and functional proximity among individuals, groups, and activities ( Büyükşahin & Aydın, 2017). The spatial configuration, defined by the arrangement of rooms, walls, doors, and separators, influences the opportunities for interpersonal visual, auditory, and behavioural interactions. Specifically, barriers, openings, street locations, and physical layouts can either facilitate or hinder social engagement.

The spatial layout design of educational spaces, such as the layout of classrooms, the placement of furniture, and the usage of common areas, can influence the power dynamics between teachers and students ( Muñoz-Cantero et al., 2016). Humans and space have a reciprocal relationship in which the physical characteristics of space, and the behaviours of the individuals who occupy it, are altered and transformed by human spatial behaviours. Educational institutions’ spatial dynamics significantly influence student and teacher interactions and learning experiences ( Yıldırım et al., 2022). Environmental psychology has established that the physical design of learning spaces may impact social relationships and student behaviour ( Gunning & Holloway, 2021). For that, Educational environments require careful design considerations to foster creativity, productivity, and positive social interactions ( Alnusairat et al., 2021; Büyükşahin & Aydın, 2017). The spatial layout and connections between different spaces in an educational environment can either encourage or discourage interactions among the people who use those places. The placement, design, and integration of places such as public rooms and informal gathering spots can influence students’ mobility and behavioural patterns, fostering or discouraging spontaneous and productive exchanges. This physical layout is critical for developing a feeling of community and facilitating creative interactions among educational environment users ( Hillier, 2007). In this sense, educational institutions’ spatial design can be purposely structured to either facilitate or inhibit social contact, which is a fundamental way that individuals use the space.

### Campus physical environment

The author reviewed existing studies on how the physical environment influences urban spaces ( Arribas-Bel & Fleischmann, 2022; Pawłowicz, 2017), with a focus on campuses ( Jansz et al., 2021; Li et al., 2023; Too & Bajracharya, 2015). They found that many design elements are essential to creating spaces where people can thrive. The more frequently a space is used, the more it becomes a place with its own identity. This transformation can happen through repeated visits from the same users or different ones, ultimately creating a sense of place. According to Pannone et al.(2019) and O’Rourke & Baldwin (2016) Placemaking and campus planning are interconnected, highlighting the importance of placemaking on campuses. On campuses, spatial organization such as openness, proximity to amenities, and integration with surrounding urban areas affects daily use and fosters creativity, knowledge exchange, and a sense of community ( Alnusairat et al., 2021).

The reviewed elements can be divided into environmental and man-made categories. Environmental factors such as thermal comfort, acoustics, and light also directly impact users’ experiences and well-being on campus. At the same time, man-made elements include seating areas, food options, art installations, and surrounding buildings ( Li et al., 2023). These elements can take many forms; for example, seating areas can be benches or chairs specifically designed for that purpose or steps repurposed as gathering spaces. Due to advances in technology and the desire for constant connectivity, Wi-Fi has also become a design element. Although intangible, concepts like cleanliness and safety significantly impact people’s comfort and sense of belonging in a space. Studies of international campuses investigated by Alnusairat et al. (2021), Eppinga et al. (2020), Jansz et al. (2021), and O’Rourke and Baldwin (2016). There are notable differences between them and many public and private universities in Bahrain. These differences include access hours, personnel, layout, and campus life. These factors can significantly influence a student’s lifestyle and perception of their university.

## Methods

This study investigates the impact of physical elements in a university-built environment on students’ social interactions. It employs various methods, including on-site observations, student surveys, informal interviews, and data visualization tools like space syntax.

To guide these methods, a literature review was conducted to identify key physical elements studied in previous research. The student gathering spots are first collected on a drawn map. During this stage, selected students were also asked to hand-draw their paths and mark nodes. The paths were mainly from the class to the cafeteria, representing their coffee break time to determine their movement (integration), then from the cafeteria to another point of their interest, and finally to the point of exit from the campus. The second and third paths are considered through movement (choice/betweenness).

To evaluate the quality of social spaces on the campus, the users were surveyed through a digital questionnaire on Google Forms. In some cases, brief follow-up interviews were conducted while students completed the survey. Participants fill out the surveys on campus to increase engagement and make the questions more relatable. The questionnaire consisted of two main parts. The first part comprises four items, primarily gathering demographic data on whether students spent their break time on campus. The second and third parts comprise 14 items, featuring a questionnaire that assessed the sensory experiences and physical features of the campus environment using a five-point Likert scale to evaluate their overall appearance and sensory attributes. Statistical analysis was then performed using SPSS to examine the relationship between the physical characteristics of the campus social spaces and students’ perceptions of their quality. In this study, high or low perception will be determined by comparing the weighted average value (the sum of the mean scores for the items divided by the total number of items).

Additionally, AutoCAD is used to create digital versions of the maps, and DepthmapX is used to analyse the paths using space syntax theory. This theory helps analyse how people move through spaces. By understanding movement patterns, planners can design areas that promote social interaction. It also identifies key areas, known as nodes, where interactions are likely to happen. These nodes can be strategically designed to boost community engagement. Then, the results of these analyses will be compared to the students’ drawn paths.

### Setting: Ahlia University, Bahrain

**Figure 2.**
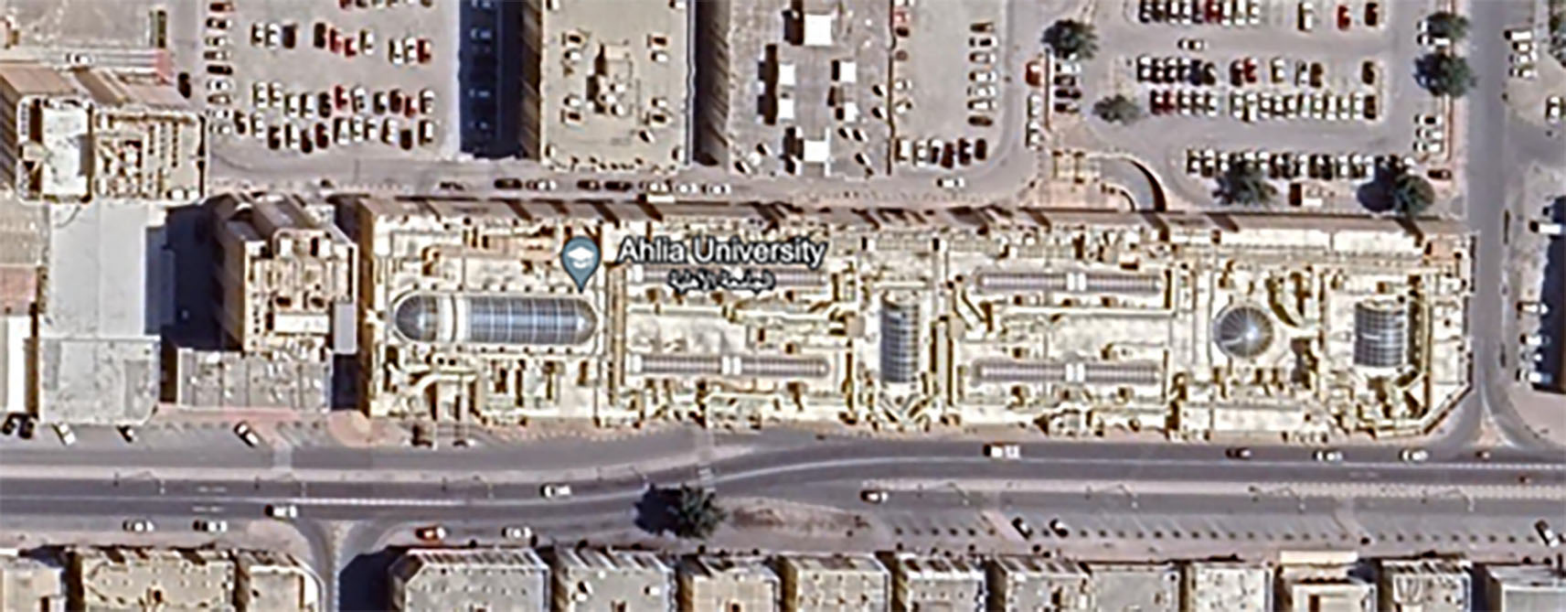
Ahlia University Campus Satellite image. Source: Intercepted by Google Maps.

Ahlia University is Bahrain’s first private higher education institution, launched in 2001. The university campus is situated within a shopping mall – GOSI complex - in the capital city, Manama, offering a unique blend of education and retail, giving students easy access to a wide range of facilities and resources ( Figure 2).

**Figure 3.**
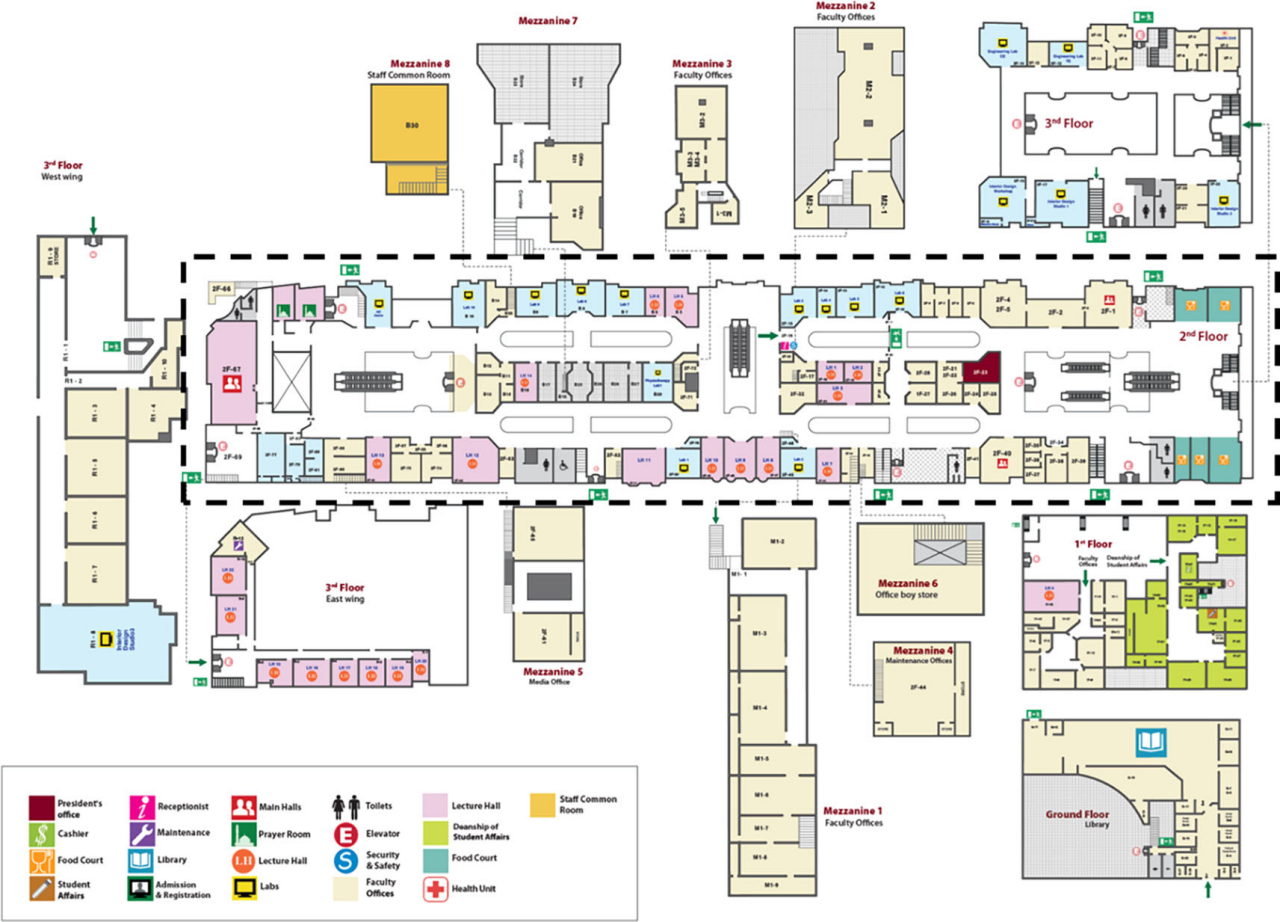
A map of the Ahlia University campus displaying various zones and their functions. Source: Ahlia University Administrative Services Directorate.

The longitudinal campus occupies the complex’s first, second, and third floors, creating a physical separation between educational and retail facilities on the ground floor ( Figure 3). Yet, visual access is maintained and positively enhances the wayfinding and navigation. Ahlia University has undergone significant transformations since its inception, driven by the increase in student numbers and purposeful renovations that have created additional spaces for learning and administrative facilities. Some of these facilities were placed in newly constructed mezzanines. These spaces were designed for students, educators, and administrative staff to use and interact with; thus, it is critical that they efficiently serve their intended function. It has been observed that the newly proposed spatial layout offers different types of path and space relationships, such as: pass by spaces, pass through spaces, terminate in a space, or reject space ( Dursun, 2007). Ahlia University classrooms are spread out through different zones, making it easier for students to cross other paths individually or with one another, increasing the sense of inclusion and the possibility of collegiality ( Alnusairat et al., 2021; Gunning & Holloway, 2021). These arrangements present users with a variety of scenes, such as being connected or disconnected from active spaces and welcomed or rejected based on the level of openness and enclosure of these spaces. As design is all about experimentation and probing, this approach allows for a range of possibilities ( Yıldırım et al., 2022). For the sake of this study, only the first floor will be considered for analysis as it is the main floor that represents most of the learning and social facilities anticipated to be accessed by students.

University campuses have complex social and spatial layouts that shape and impact the patterns of interactions of students ( Gunning & Holloway, 2021; Hanan, 2013). Educational institutions’ spatial layout and architectural design may have a significant impact on the nature and frequency of formal and unintended student interactions ( Büyükşahin & Aydın, 2018). Researchers have found that well-designed common areas, such as corridors, foyers, and libraries, can foster social interaction and help students develop a sense of belonging and comfort. Repurposing existing educational facilities, such as transforming unused zones into more flexible and attractive collaboration zones, can help students develop a sense of community and engage in social activities.

**Figure 4.**
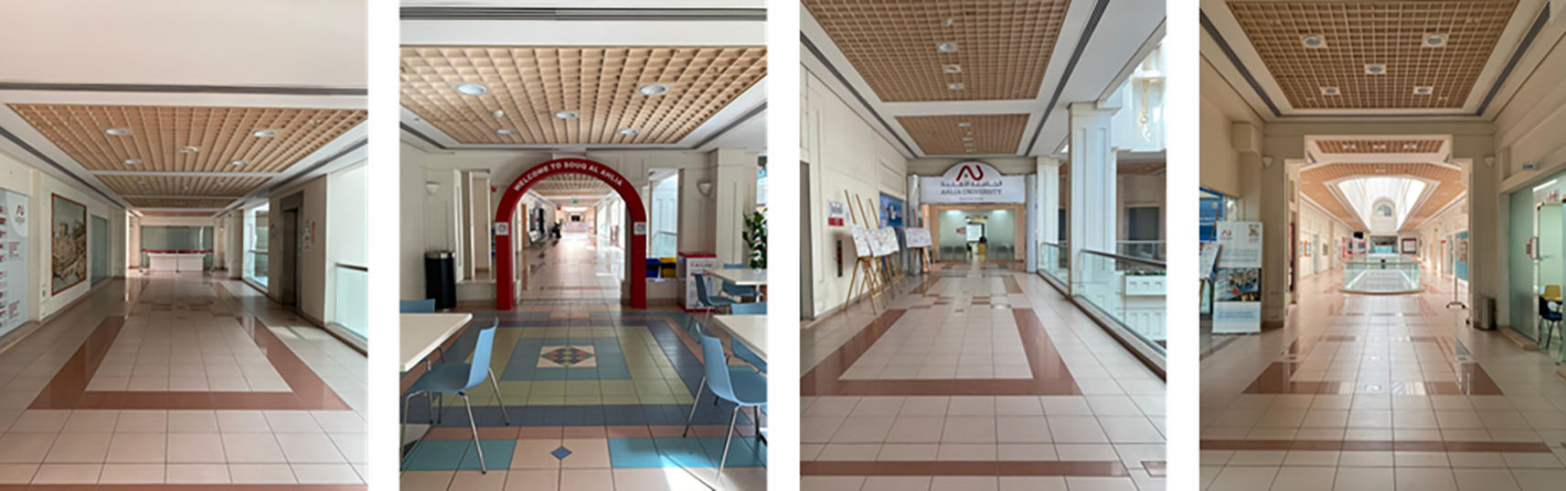
A representation of the first-floor hallway design at Ahlia University. Source: Author.

The existing campus is completely enclosed and lacks accessible green spaces and an outdoor landscape due to the original nature of the building, which is a shopping mall, which may impact the students’ mental health and well-being. ( Dooris et al., 2021). The available social spaces for student gatherings on campus are limited, primarily consisting of the cafeteria, student lounge, and some dispersed seating areas in the hallways. Figure 4 illustrates different shots of the university’s first-floor hallways. The shots were taken when no students were present to ensure personal privacy protection. In regular operating hours, these hallways are used for gathering, navigation, and transition. However, they are poorly designed and planned, with few benches distributed along the hallway and limited capacity seating in the cafeteria.

### Participants

All participants in the study were undergraduate students at Ahlia University. Students gladly participated in the questionnaire once they knew it was related to their campus, hoping the research would lead to future improvements. In the survey, the demography section revealed the students’ gender and year. A total of 139 questionnaires were distributed via Microsoft Teams. However, only 131 of the questionnaires were considered due to their realistic and reliable answers with a reliability coefficient of 0.91. This high coefficient shows strong consistency in responses, confirming the validity of the findings. The use of standardized questions and a clearly defined target population further improves the reliability of the data.

The final sample was 79.4 percent females and 20.6 percent males. A total of 62.6 percent of the participants are students in the third and fourth years, while 20.6 percent are enrolled in the first and second years, and only 16.8 percent are students who have been enrolled for more than four years. The second part was used to determine the amount of time students spent interacting on campus while not in class (breaks between lectures), most respondents confirmed that they have a one-hour break between their lectures, representing 59.5 percent, followed by 33.6 percent who have on average two to three hours break between their lectures. The remaining students have more than three hours’ break.

## Results and Discussion

**Figure 5.**
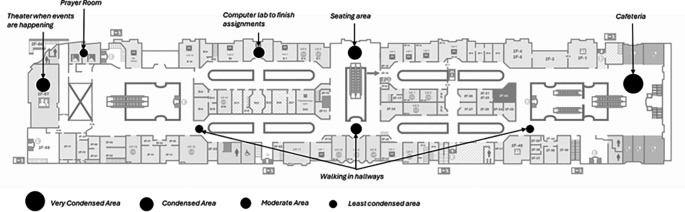
A map illustrating the main areas where students gather socially on the first floor before and after classes. Source: Author.

The author gathered a sample of 15 undergraduates to gain insights into their use of campus social spaces during free time before and after classes. The author then mapped the participants’ responses to identify their preferred locations on the university grounds. These areas were then categorized into four places: very condensed, condensed, moderate, and less condensed, as shown in Figure 5. Most students said that the cafeteria is their favorite place, followed by a few benches along the hallway, and some preferred gathering at the student lounge on the third floor.

**Figure 6.**
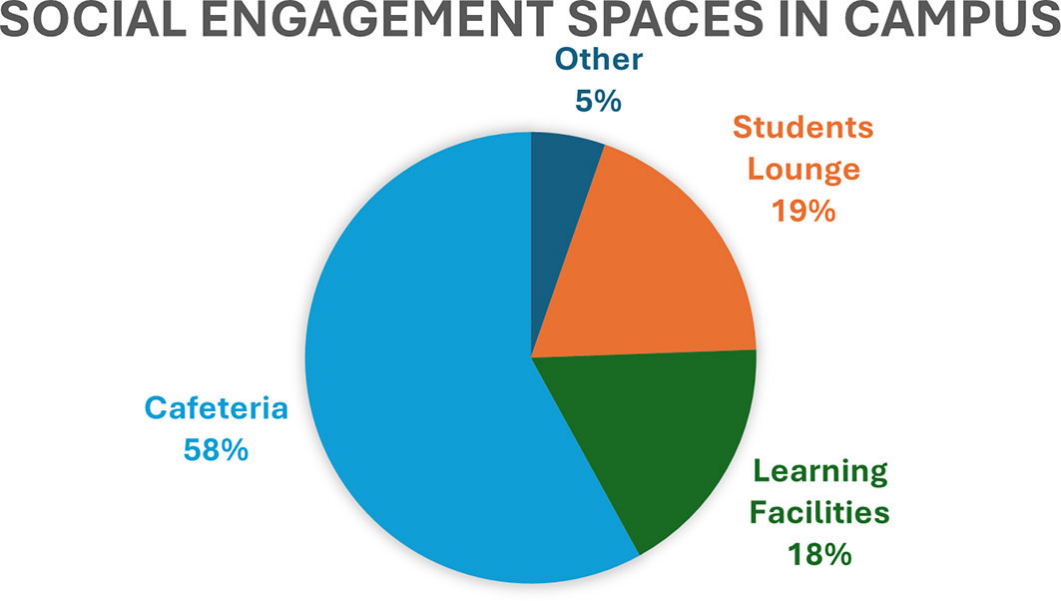
A graphical depiction highlighting the key locations on campus where students gather socially before and after classes (n = 131). Source: Author.

A graphical display is used to illustrate the spaces commonly frequented by respondents during their free time before and after classes, as detailed in the first part of the questionnaire ( Figure 6).

The visual representation indicates that the cafeteria was the campus space most frequently visited by students for social gatherings before and after their academic activities, consistent with the spatial map provided in Figure 4. Despite the gathering locations shown on the map and in the illustrated chart, many students also spent their free time in the library study rooms on the ground floor to complete academic tasks. Additionally, several male students reported spending their free time on the third floor, which offered recreational amenities such as table tennis, card tables, and a student lounge. According to the follow-up interview findings, many female students expressed a sense of exclusion from the third floor, which was perceived as designated primarily for male students. Some participants further reported that the noise and disturbances originating from this floor negatively impacted their ability to concentrate in the classroom or even just to relax.

Regarding the respondent’s opinion on sensory effects in general, within the spaces they use for social interaction, as shown in Table 1, the high or low perception will be determined by comparing the weighted average value to the mean. In the case of sensory effects, the weighted value is 3.28. The data analysis indicates that most respondents perceived the university campus as providing a quiet, safe, and clean environment. On the other hand, the respondents had a low perception of sensory factors related to comfort and entertainment.

**Table 1.** Respondent’s opinion on sensory effects (n = 131). Source: Author.

Sensory effects	Very bad	Bad	Moderate	Good	Very good	Mean	Std. deviation	Decision
	(%)	(%)	(%)	(%)	(%)			
Comfortable	12 (9.2%)	21 (16%)	44 (33.6%)	40 (30.5%)	14 (10.7%)	3.1756	1.11273	Low perception
Entertaining	28 (21.4%)	35 (26.7%)	42 (32.1%)	18 (13.6%)	8 (6.1 %)	2.5649	1.15091	Low perception
Quietness and safety	11 (8.4%)	16 (12.2%)	32 (24.4%)	34 (26%)	38 (28%)	3.5496	1.26012	High perception
Clean	10 (7.6%)	8 (6.1%)	17 (13%)	46 (35.1%)	50 (38.2%)	3.9008	1.20163	High perception
General evaluation	16 (12.2%)	20 (15.3%)	41 (31.2%)	28 (19.8%)	26 (19.8%)	3.2137	1.27102	High perception

According to one of the survey questions, some students reported that they do not prefer to spend their break time on campus, and the answers were as stated below:

*“There are no activities, and the cafeteria is always full of Talabat (food delivery service), and there is a bad smell," said by a Female student.*

*"There is no place for me to be comfy and quiet, or different restaurants to choose from and enjoy," said by a Female student.*

*"Because there are no comfortable places in the university, no good restaurants, and no activities,", said by a male student.*

*“Due to the lack of dedicated break rooms that allow for comfortable sitting or relaxation, in addition to the lack of basic amenities such as quiet, air-conditioned spaces, and the limited or sometimes unavailable food options, staying on campus during free time makes it uncomfortable and impractical.”, said by a Female student.*

In summary, comfort and entertainment aspects received low perceptions, highlighting areas for improvement. The reasons behind their low perceptions appear to be related to the cafeteria’s seating quality, as most students expect to spend their free time between and after classes, as well as the limited food options. Additionally, the university lacks recreational facilities and rest or break rooms.

The connection between comfort and entertainment, as seen by students on a university campus, plays a key role in shaping their overall experience. Comfortable spaces, such as well-designed lounges and study areas, encourage students to participate in entertainment activities, including social events and recreational facilities ( Büyükşahin & Aydın, 2017). When students are relaxed, they’re more likely to succeed, get involved in campus life, and build a sense of community and belonging. This relationship suggests that universities should prioritize creating comfortable spaces to enhance student engagement and well-being, ultimately enriching both the academic and social aspects of the experience ( Alnusairat et al., 2021; Li et al., 2023).

From reviewing the campus physical features measurements, as shown in Table 2, the high or low perception will be determined by comparing the weighted average value to the mean. In the case of campus physical features, the weighted value is 3.07. The data analysis indicates that respondents seem to have a high perception of the seating at learning facilities (i.e., classrooms, computer labs), access to natural light (i.e., skylights in campus corridors), which resulted in a high perception of boundaries between the multiple facilities happening at the campus, and clear paths to navigate from zone to another. A well-designed signage and wayfinding system supports the latter.

**Table 2.** Respondent’s opinion on campus physical features (n = 131). Source: Author.

Campus physical features	Very bad	Bad	Moderate	Good	Very good	Mean	Std. deviation	Decision
	(%)	(%)	(%)	(%)	(%)			
Seating at learning facilities	16 (12.2%)	22 (16.8%)	46 (35.1%)	30 (22.9%)	17 (13%)	3.0763	1.18723	High perception
Natural light	9 (6.9%)	10 (7.6%)	31 (23.7%)	40 (30.5%)	41 (31.3%)	3.7176	1.18500	High perception
Clear boundaries	9 (6.9%)	13 (9.9%)	25 (19.1%)	47 (35.9%)	37 (28.2%)	3.6870	1.18376	High perception
Paths	10 (7.6%)	17 (13%)	18 (13.7%)	43 (32.8%)	43 (32.8%)	3.7023	1.26305	High perception
Gaming and recreational facilities	51 (38.9%)	31 (23.7%)	32 (24.4%)	11 (8.4%)	6 (4.6%)	2.1603	1.16894	Low perception
Social spaces	26 (19.8%)	27 (20.6%)	40 (30.5%)	22 (16.8%)	16 (12.2%)	2.8092	1.27771	Low perception
Reliable Wi-Fi network	35 (26.7%)	24 (18.3%)	35 (26.75%)	23 (17.6%)	14 (10.7%)	2.6718	1.32690	Low perception
Seating in the cafeteria	25 (19.1%)	22 (16.8%)	43 (32.8%)	31 (23.7%)	10 (7.6%)	2.8397	1.20777	Low perception
General evaluation Physical	16 (12.2%)	21 (16%)	52 (39.7%)	24 (18.3%)	18 (13.7%)	3.0534	1.17874	High perception

On the other hand, nearly 90% of the respondents had a low perception of the gaming and recreational facilities, and almost 70% reported a low perception of the availability of social spaces for relaxation and conversation. These concerns were also mentioned during the follow-up interview session. Here are some of the students’ comments:

*“I do not find campus activities appealing.”, said by a male student.*

*“There are no comfortable sitting areas in the university, and there are no entertaining areas for students”, said by a female student.*

*“Not enough entertainment facilities, especially for females”, said by a female student.*

*“The internet is very weak, I have to rely on my cellular data”, said by a male student.*

A closer look at the first statement reveals a lack of interest in the available extracurricular activities, which may indicate a gap between what the university offers and what students find engaging. A similar situation was highlighted by Alnusairat et al. (2021), who focused on integrating social and contextual factors to create meaningful spaces in universities. The second comment highlights the lack of physical spaces that encourage relaxation and social interaction, both of which are essential for student well-being, as stated in previous studies ( Deslauriers et al., 2019; Hanan, 2013). The third quote highlights a perceived gender imbalance in entertainment facilities, suggesting that female students may feel underserved in this area. The last quote suggests that the internet service provided is inconsistent or inadequate for their needs, impacting their ability to study or participate in online activities.

Furthermore, nearly 70% of respondents had a low perception of the Wi-Fi network’s quality, which can negatively impact students in several ways, including academic challenges like accessing resources and communicating instantly with professors and classmates. Additionally, it is expected to decrease students’ engagement in online activities and restrict their access to learning platforms. Similarly, Harrath & Alobaidy (2016) identified the negative impacts of Wi-Fi services on students’ academic performance and social engagement. Unexpectedly, nearly 69% of the respondents showed a low perception of the cafeteria seating, yet 58% of the students indicated that they spent their breaktime between and after their lectures in the cafeteria, as shown in Figure 6. The discrepancy between the low perception of cafeteria seating and the high percentage of students spending their break time there raises several intriguing points for consideration, including, but not limited to, the seating being uncomfortable or inadequately designed, leading to dissatisfaction despite being used frequently, the cafeteria may become overcrowded during peak times, making it less enjoyable, even if students choose to spend time there, and most probably students may have had higher expectations for the cafeteria experience, which were not met, leading to a low perception score.

To analyse the sensory evaluation, the campus’s physical features were assessed to determine their impact on sensory experiences using SPSS. As demonstrated in Table 3, well-defined boundaries, seating arrangements, and social areas have a significant impact. Features like natural light and reliable Wi-Fi also play a role, especially in enhancing the entertainment aspect. The most critical factors contributing to an entertaining experience are gaming, recreational facilities, and social spaces. Thus, expanding areas for social interaction is vital to enhance overall comfort and entertainment. Additionally, ensuring reliable internet access is crucial, as it plays an essential role in the overall entertainment experience.

**Table 3.** Correlation between sensory effects and physical features (n = 131). Source: Author.

		Sensory effects				
Physical features		Comfortable	Entertaining	Quietness	Clean	General evaluation
Seating at learning facilities	r	0.566 **	0.374 **	0.522 **	0.523 **	0.504 **
	p	0.000	0.000	0.000	0.000	0.000
Natural light	r	0.225 **	0.129	0.285 **	0.515 **	0.332 **
	p	0.010	0.141	0.001	0.000	0.000
Clear boundaries	r	0.468 **	0.379 **	0.642 **	0.713 **	0.515 **
	p	0.000	0.000	0.000	0.000	0.000
Paths	r	0.404 **	0.355 **	0.500 **	0.644 **	0.366 **
	p	0.000	0.000	0.000	0.000	0.000
Gaming and recreational facilities	r	0.434 **	0.607 **	0.378 **	0.170	0.381 **
	p	0.000	0.000	0.000	0.052	0.000
Social spaces	r	0.586 **	0.581 **	0.472 **	0.348 **	0.556 **
	p	0.000	0.000	0.000	0.000	0.000
Reliable Wi-Fi network	r	0.456 **	0.480 **	0.472 **	0.250 **	0.389 **
	p	0.000	0.000	0.000	0.004	0.000
Seating in the cafeteria	r	0.588 **	0.497 **	0.544 **	0.413 **	0.534 **
	p	0.000	0.000	0.000	0.000	0.000
General evaluation Physical	r	0.673 **	0.584 **	0.555 **	0.427 **	0.706 **
	p	0.000	0.000	0.000	0.000	0.000

r: Pearson coefficient.

** Statistically significant at p ≤ 0.01.

Furthermore, the correlation analysis of sensory effects and physical features reveals significant relationships related to personal attraction factors, such as individual preferences, similarity, familiarity, and physical proximity. For instance, the strong correlation between seating at learning facilities and comfort (r = 0.566) and entertainment (r = 0.374) highlights how personal preferences in seating enhance individual experiences. Social spaces, with a strong correlation (r = 0.586 for comfort and r = 0.581 for entertainment), promote interactions among individuals with similar interests, fostering a sense of community. Familiarity also plays a key role, as shown by the moderate correlation of natural light (r = 0.225 for comfort), which can make spaces feel more inviting, while clear boundaries (r = 0.642 for quietness) contribute to a sense of security and comfort. Physical proximity is important as well, with pathways showing a moderate correlation (r = 0.404) with comfort, indicating that well-designed pathways improve accessibility to facilities and encourage engagement. The strong correlation between gaming and recreational facilities (r = 0.607 for entertainment) further emphasizes that proximity to these areas can facilitate social interactions. Overall, the analysis suggests that thoughtful design of physical features—focusing on comfort, clarity, and accessibility can significantly enhance personal attraction factors, creating environments that foster connection, engagement, and overall satisfaction among individuals.

**Figure 7.**
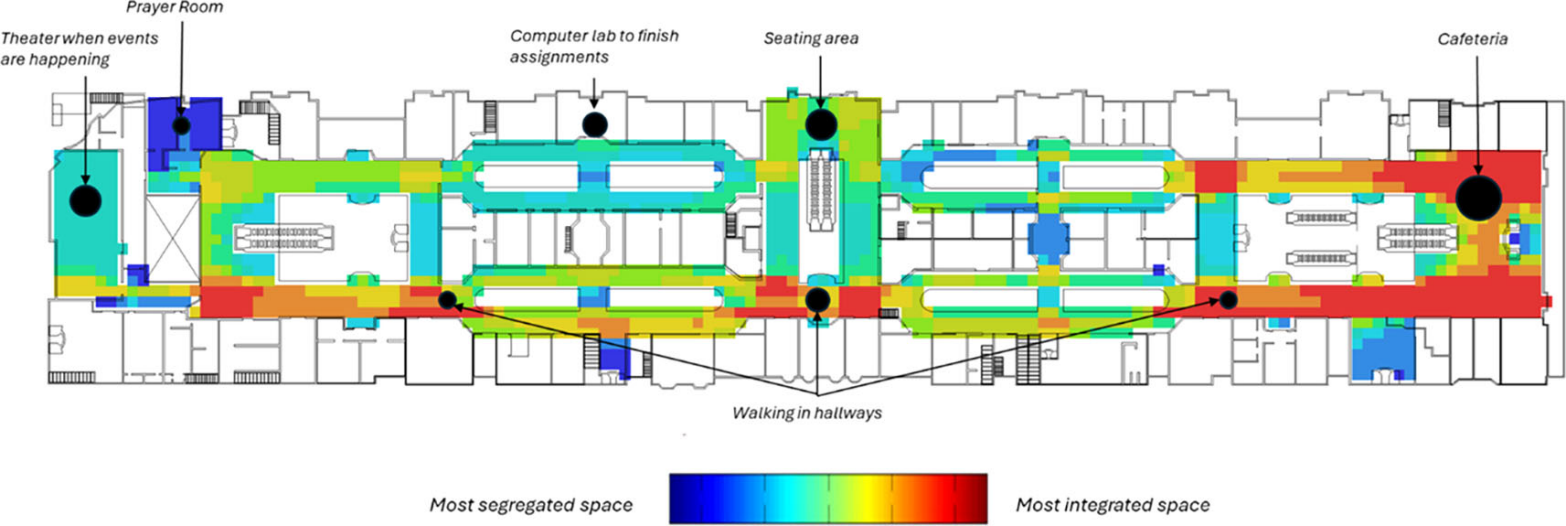
Visibility Graph Analysis (VGA) employed to forecast student behavioral patterns. Source: Adopted using DeptmapX software.

**Figure 8.**
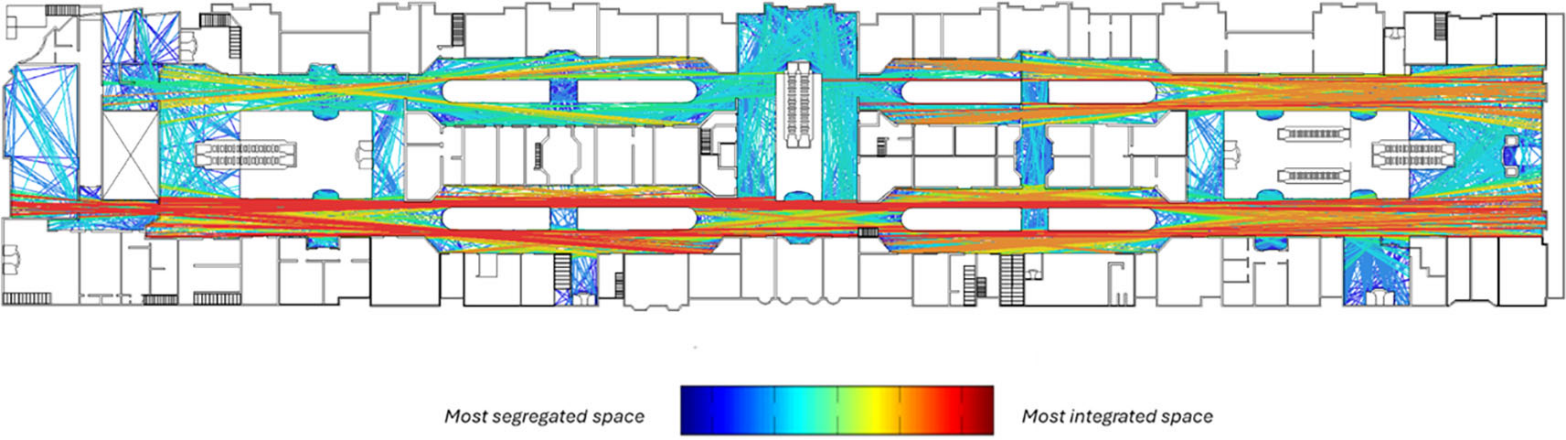
Axial Map Analysis (Integration) is utilized to illustrate the continuous perception of visibility and permeability experienced by observers as they navigate the building. Source: Adopted using DeptmapX software.

The results of the mostly closed entrances plan are shown in Figure 7 of the visibility graph analysis. The red colour represents the most integrated level, and the blue colour represents the most segregated. Visibility Graph Analysis (VGA) was used to estimate and predict the behavioural patterns of students, and to compare their points of interest as indicated in Figures 5 and 6. The map shows that the cafeteria is the most connected space, with a numerical value of 261, while the prayer room is the most segregated space. The axial analysis shown in Figure 8 is used to represent how far an uninterrupted impression of visibility and permeability observers can have as they move about the second floor and look at a distance in various directions. The result revealed that the cafeteria and its adjacent corridors are the most integrated areas within the layout, with a numerical value of 14.19. The results from both the visibility map and axial analysis confirm the data collected from the questionnaire and the follow-up interview, suggesting that designing spaces with comfort, clarity, and accessibility in mind can enhance personal attraction factors. For instance, seating arrangements that encourage socialization can lead to stronger community bonds. Also, incorporating familiar design elements can help individuals feel more at ease, fostering positive interactions.

## Conclusion and Recommendations

The study of movement on campus, along with the analysis of gathering spaces, is crucial for understanding students’ needs. Creating a suitable physical environment for interaction encourages students to spend more time on campus. In this research, the movement findings cannot be generalized to the entire campus, as the focus was mainly on the first floor—the most active level with most learning and social spaces expected to be used by all students regardless of their studied programs. The first floor also influenced movement patterns across the whole campus. It was observed that students more satisfied with campus features tend to spend more time engaging with others, while those dissatisfied feel less ownership, less capable of making changes, and usually spend less time on campus. Spaces with more seating, such as the cafeteria and hallways, were found to be more attractive. Sitting areas were commonly present in nearly all gathering spaces. Food and beverage options are highly valued by students, being seen as attractive features and key factors encouraging longer campus stays. Although the cafeteria is centrally located and easily accessible, as shown by movement and space syntax analysis, it still lacks sensory qualities like comfort and entertainment. This indicates that adding social rooms, activity rooms for gaming and recreation, along with diverse food options, could expand movement patterns and boost campus activity. This research covers only a small part of the larger effort to develop effective strategies for broader design goals. This is an active, evolving field that constantly generates new ideas and involves diverse stakeholders. Future designs and approaches are expected to emerge as campus spaces expand or when moving to a new, promising campus, making the roles of architects, decision-makers, and planners even more vital.

From a broad perspective, campus design and social interaction spaces are crucial and require careful planning through specific recommendations, guidelines, and measures. Building on the current study, a targeted set of recommendations is proposed: incorporating end-user opinions into the design process enhances satisfaction; scientific analysis using Space Syntax theory should inform decisions in campus design, particularly for expanding campuses, social spaces, and activity zones. Safety and security considerations should be integrated into decision-making processes. Utilizing computer simulation tools can support decision-making and should be routinely employed. Additionally, it is vital to continually gather and consider users’ satisfaction and feedback in every design stage.

## Ethics and consent

Ethical Approval was granted by the Ahlia University Research Ethics Framework (AUREF) under the University Council Decision No.: UC/1872/06/2018-19 and granted on 22 March 2024. Ethical approval stated that the requirements for data availability in no way override the right to confidentiality and privacy of individuals or organisations who are the subjects of research.

## Participant consent

Informed consent was obtained from all participants digitally through the online survey. Once again, informed consent was sought before each follow-up interview, as participants were briefed on the study’s purpose and invited to provide their informed consent, having already given their consent digitally through the online survey.

## Data Availability

Figshare: Comfort and Entertainment Under Examination: The Effects of Campus Design on Student Social Interaction – Findings from Bahrain, https://doi.org/10.6084/m9.figshare.29827673.v1, Al Saffar, May (2025). The project contains the following underlying data:
• Data.xlsx • Data.sav Data are available under the terms of the Creative Commons Attribution 4.0 International License (CC-BY 4.0). Figshare: Comfort and Entertainment Under Examination: The Effects of Campus Design on Student Social Interaction – Findings from Bahrain, https://doi.org/10.6084/m9.figshare.29827673.v1, Al Saffar, May (2025). The project contains the following underlying data:
• Questionnaire.doc This content is available under the terms of the Creative Commons Attribution 4.0 International License (CC-BY 4.0).
