# Supplementary material for: Exploring Comfort and Entertainment: The Impact of Campus Design on Student Social Interaction — Insights from Bahrain
Source: F1000Res. 2025 Sep 4;14:870. [Version 1] doi: 10.12688/f1000research.167517.1 (PMC12521896; doi:10.12688/f1000research.167517.1)
Supplement: Peer review history

# Reviewer report: Reviewer response for version 1

**DOI:** 10.5256/f1000research.184636.r412853

**Published:** 2025-10-14

**Authors:** Dalia Eldardiry

**Affiliations:** 1 Imam Abdulrahman Bin Faisal University, Dammam, Saudi Arabia

This paper offers a timely and insightful investigation into how campus design influences student social interaction at Ahlia University in Bahrain. The research is particularly compelling as it examines the dimensions of comfort and entertainment within student spaces. It employs a robust mixed-methods approach that thoughtfully blends surveys, interviews, and space syntax analysis. The manuscript is well-written, firmly grounded in theory, and concludes with practical, actionable recommendations. Its originality shines through its unique context: a university situated within a shopping mall, providing a fresh perspective on placemaking, student well-being, and engagement within the distinctive environment of the Gulf region.

 Key Strengths:

- Clarity and Relevance: The paper's focus is clear and highly relevant. It addresses a critical question for educational quality: how physical design either fosters or hinders the social interaction and comfort vital to academic performance and student well-being.
- Original Context: The unusual mall-based campus setting is not just a curiosity but a fascinating case study that generates insights far beyond conventional campus planning, especially valuable in a region where such research is scarce.
- Methodological Accuracy: Integrating multiple methods, surveys, interviews, observations, and space syntax analysis results in a comprehensive and convincingly triangulated study. The high reliability of the survey data (Cronbach’s α = 0.91) further bolsters confidence in the findings.
- Transparency and Open Science: Sharing the datasets and research instruments via Figshare is a commendable practice that enhances the study's replicability and aligns with the best open science standards.
- Practical Application: The transition from analysis to actionable recommendations—such as improving seating, enhancing recreational facilities, and addressing gender dynamics—is exceptionally strong. This makes the paper immediately useful for university administrators and architects.

**Areas for Development and Suggestions:**

 While the paper is undoubtedly strong, a few refinements could amplify its impact:

- Policy Connections: The findings could be more powerfully framed by connecting them to broader institutional or national higher education policy frameworks. Adding a dedicated “Policy Implications” section to discuss alignment with, for example, Bahrain’s Higher Education Council strategies or international guidelines from bodies like UNESCO or the WHO would significantly broaden its relevance.

This paper represents a substantial and original contribution to the field, successfully marrying methodological rigor with practical relevance. It is already a strong piece of work. With a stronger policy dimension, a deeper exploration of gender inclusivity, and more comprehensive methodological reporting, it can become even more impactful. These refinements would elevate the study's value for scholars, maximize its utility for practitioners in campus planning, and ensure it resonates with policymakers in higher education.

Is the work clearly and accurately presented and does it cite the current literature?

Yes

If applicable, is the statistical analysis and its interpretation appropriate?

Yes

Are all the source data underlying the results available to ensure full reproducibility?

Yes

Is the study design appropriate and is the work technically sound?

Yes

Are the conclusions drawn adequately supported by the results?

Yes

Are sufficient details of methods and analysis provided to allow replication by others?

Yes

Reviewer Expertise:

Urban Design, Design Education

I confirm that I have read this submission and believe that I have an appropriate level of expertise to confirm that it is of an acceptable scientific standard.

## References

[1] : Exploring Comfort and Entertainment: The Impact of Campus Design on Student Social Interaction — Insights from Bahrain. *F1000Research* .2025;14: 10.12688/f1000research.167517.1 10.12688/f1000research.167517.1 40657179

# Reviewer report: Reviewer response for version 1

**DOI:** 10.5256/f1000research.184636.r412851

**Published:** 2025-09-30

**Authors:** Islam Elghonaimy

**Affiliations:** 1 University of Bahrain, Zallaq, Bahrain

This article presents a well-structured mixed-methods study investigating the influence of campus design on student social interaction at Ahlia University, Bahrain. The research question is relevant, and the methodology—combining surveys, observations, and space syntax analysis—is appropriate and rigorously applied. The manuscript is clearly written, logically organized, and effectively situates the study within existing literature on environmental psychology and spatial design.

 The findings are robust and clearly presented. They demonstrate that while students primarily gather in central areas like the cafeteria, their perceptions of comfort and entertainment are low due to insufficient seating, limited recreational facilities, and unreliable Wi-Fi. Using statistical analysis and space syntax (Visibility Graph and Axial Analysis) provides strong, data-driven support for these conclusions, convincingly linking specific physical features to sensory experiences and social engagement. The inclusion of direct student quotes enriches the qualitative findings.

 In summary, this is a study that makes a concrete contribution to the discourse on learning environments. Its practical recommendations are well-founded in the data. The main area for improvement would be to address the limitations of the single-case study design more explicitly in the discussion.

Is the work clearly and accurately presented and does it cite the current literature?

Yes

If applicable, is the statistical analysis and its interpretation appropriate?

Yes

Are all the source data underlying the results available to ensure full reproducibility?

Yes

Is the study design appropriate and is the work technically sound?

Yes

Are the conclusions drawn adequately supported by the results?

Yes

Are sufficient details of methods and analysis provided to allow replication by others?

Yes

Reviewer Expertise:

Urban design, urban development, sustainable development, landscape architecture

I confirm that I have read this submission and believe that I have an appropriate level of expertise to confirm that it is of an acceptable scientific standard.

# Response

**Published:** 2025-10-04

**Authors:** May Al-Saffar

Dear Prof. Elghonaimy,

 Thank you very much for your positive feedback and for approving my submission. I truly appreciate the time and effort you dedicated to reviewing my work.

 Regards,

 May
